# A Preliminary Report on the Effects of Daridorexant in Patients with Comorbid Insomnia and Substance Use Disorders

**DOI:** 10.3390/ph18030378

**Published:** 2025-03-06

**Authors:** Marco Di Nicola, Maria Pepe, Lorenzo Bonomo, Miriam Milintenda, Isabella Panaccione, Roberto Brugnoli, Gabriele Sani

**Affiliations:** 1 Department of Neuroscience, Section of Psychiatry, Università Cattolica del Sacro Cuore, L.go Francesco Vito 1, 00168 Rome, Italy; 2 Department of Psychiatry, Fondazione Policlinico Universitario Agostino Gemelli IRCCS, L.go Agostino Gemelli 8, 00168 Rome, Italy; 3 Department of Mental Health, ASL Roma 1, Piazza Santa Maria della Pietà 5, 00135 Rome, Italy

**Keywords:** DORA, sleep disorders, dual diagnosis, craving, personalized medicine

## Abstract

**Background**. Sleep disturbances are frequent in patients with substance use disorders (SUDs) and are associated with craving and addiction relapses, leading to increased clinical severity and detrimental outcomes. Daridorexant, a selective dual orexin receptor antagonist, has been approved for persistent insomnia disorder (ID), but specific insights on patients with SUDs are lacking. **Methods**. This observational, retrospective study investigated the effects of a three-month treatment with daridorexant (50 mg/day) in 41 outpatients with comorbid IDs and SUDs. Improvement in subjective sleep measures, assessed with the Insomnia Severity Index (ISI) and subjective total sleep time, was the primary outcome measure. Changes in anxiety and depression symptoms, quality of life, clinical global severity, and craving were also investigated through the following: Hamilton Anxiety and Depression Rating Scale; Five-item World Health Organization Well-Being Index; Clinical Global Impression Severity Scale; Visual Analog Scale for Craving. **Results**. All sleep outcomes significantly improved throughout treatment, which was generally safe and well tolerated, with mild and transient drowsiness and sluggishness reported in 21.1% of patients. Similar improvements were observed in psychopathology, quality of life, and craving, and positive correlations were found among ISI scores and anxiety/depression symptoms and craving. An abstinence rate (i.e., absence of any substance use, regardless of the amount, throughout treatment) of 65.8% was also detected at the endpoint. **Conclusions**. These preliminary findings suggest that daridorexant might represent a promising tool for treating insomnia in patients with SUDs. Identifying interventions effectively targeting insomnia with a good safety/tolerability profile in SUDs is crucial to achieve remission and full functional recovery.

## 1. Introduction

Insomnia is defined as a difficulty initiating or maintaining sleep, with subsequent poor sleep quality and impaired daytime functioning and quality of life, and might occur as an independent disorder or in association with other physical/mental illnesses [1]. It represents a significant public health burden affecting almost 10% of the general population and up to 40–50% of individuals with mental illness [2]. In psychiatric settings, insomnia may be a risk factor, a transdiagnostic symptom, or a comorbid condition and has been associated with more severe psychopathology, impaired remission, and worse outcomes, regardless of its presentation [2,3].

Sleep disturbances are reported in most mental diseases and are widespread in patients with substance use disorders (SUDs), with rates from 30% to 80% depending on the main substance of abuse [3,4]. Conversely, people suffering from insomnia tend to be more prone to substance use, suggesting a bidirectional relationship [5]. Sleep problems may occur in any phase of the addiction and are associated with increased craving, relapses, and overall severity [6].

Targeting insomnia has become a focus of treatment, as sleep improvements have been correlated with reductions in psychiatric symptoms, such as depression and anxiety, also in patients with SUDs [7,8]. Nevertheless, most medications used for treating insomnia, such as Z-drugs and benzodiazepines, may be contraindicated in these patients because of the risk of drug–drug interactions as well as misuse, abuse, and addiction [6]. Evidence indicates that the hypnotic efficacy of benzodiazepines diminishes after one to two weeks of continuous intake, promoting tolerance and dependence [9], while chronic use can result in withdrawal symptoms upon discontinuation including rebound insomnia, anxiety, and irritability, which further complicate clinical management [10,11]. Individuals with a history of substance use exhibit a heightened susceptibility to these neuroadaptive processes and, consequently, an increased risk of developing dependence on similar compounds [12,13]. Moreover, prolonged use has been associated with significant impairments of both physical and mental functions, as well as with an increased risk of overdose, particularly if combined with other depressant substances [14,15]. Thus, managing insomnia in SUDs can be challenging due to the complex interplay between sleep disturbances, psychiatric symptoms, and substance-related effects, often requiring multiple medications that further increase the likelihood of adverse effects [16,17]. Therefore, despite the high prevalence and the significant clinical implications, the treatment of insomnia disorders remains an unmet need in this vulnerable population.

The orexin system has a pivotal role in regulating the sleep–wake cycle and seems to be involved in several physiological functions, emerging as a promising therapeutic target [4]. Orexinergic neurons are primarily localized in the lateral hypothalamus and project widely throughout the neuroaxis, influencing a broad range of processes, from autonomic regulation to higher-order functions such as attention, cognition, mood, and hedonic tone [18,19]. Both animal and human studies have reported the role of the orexin system in regulating motivational activation, i.e., transforming motivationally relevant states into targeted behaviors, such as food-seeking during hunger or drug-seeking during withdrawal [20]. These effects are partly mediated by the stimulation of dopaminergic activity in brain areas involved in reward processing [21,22] with the subsequent enhancement of motivational drive. Additional actions have been reported on the GABAergic system: while direct disinhibition promotes arousal during wakefulness, the indirect modulation of dopaminergic neurons in reward-related regions further enhances dopamine release, reinforcing addictive behaviors [23,24]. Indeed, increased orexin levels have been associated with several psychiatric disorders presenting with motivational dysfunctions, including depression, anxiety, attention-deficit/hyperactivity disorder, and addiction [19].

Daridorexant, a selective dual orexin receptor antagonist (DORA) recently approved for the treatment of persistent insomnia disorder (ID), has demonstrated efficacy in improving both sleep outcomes and daytime functioning [25,26]. Most studies have evaluated these effects after one and three months of treatment, in accordance with recommendations to use the shortest effective duration and assess efficacy within the first three months before deciding whether to continue treatment, followed by regular monitoring thereafter [25,27,28]. Evidence supports its favorable safety and tolerability profile, along with a generally low potential for abuse [25,26]. The novel mechanism of action differs from traditional insomnia treatments based on modulation of GABA receptors, preserving sleep architecture and reducing the potential for abuse [16]. Moreover, although other DORAs have shown similar efficacy, they may differ in their pharmacokinetic profiles and next-day residual effects [29].

In contrast, limited research is available on the pharmacological interactions of daridorexant with psychotropic substances. Existing evidence primarily focuses on central nervous system (CNS) depressants, particularly alcohol, showing that such combinations can lead to additive effects on psychomotor performance, increase the risk of cognitive impairment and CNS depression, and should be avoided [30]. Although significant pharmaco-kinetic/dynamic interactions with stimulants or other psychotropic substances have not been described so far, research fully elucidating such interactions is needed [31].

Beneficial effects of DORAs (e.g., suvorexant) on substance-related problems have been reported by several studies, but specific evidence regarding the safety and efficacy of daridorexant in populations with SUDs remains limited [32]. Given the high prevalence of insomnia among these individuals and the risks associated with traditional treatments, investigating daridorexant as a potential therapeutic option is warranted. Therefore, aiming to address this gap, this retrospective, observational study evaluated outpatients with comorbid insomnia and substance use disorders treated with daridorexant for three months. Improvement in sleep features was the primary outcome measure. Changes in anxiety and depressive symptoms, quality of life, clinical global severity, and craving for substances were also investigated as secondary outcomes.

## 2. Results

Two-hundred sixteen patients referred for SUDs were screened for enrollment and, after removing those who did not fulfill the inclusion criteria (n = 109), presented missing data in their medical charts (n = 50), or refused to participate (n = 16), a total of forty-one patients with comorbid IDs and SUDs were included. Alcohol (28.8%), sedative–hypnotics (22.4%), cocaine (9.8%), cannabinoids (4.9%), opioids (2.4%), and multiple use (31.7%) were found as the main substances of abuse. An additional psychiatric diagnosis was present in 48.8% of the sample including anxiety disorders (28.4%), depressive disorders (25.7%), personality disorders (20.8%), bipolar disorders (16.5%), food and eating disorders (5.3%), and adult attention deficit/hyperactivity disorders (3.3%).

At baseline, 68.3% of the sample (n = 28) showed insomnia that lasted more than 12 months. All sociodemographic, clinical, and psychometric information at baseline are summarized in Table 1.

Concomitant pharmacological treatments were as follows: anticonvulsants/mood stabilizers (80.5%), antidepressants (75.6%), anti-craving drugs (41.5%), antipsychotics (34.1%), and anxiolytics (31.7%). Insomnia treatments ongoing at baseline (i.e., low-dose quetiapine, promazine, trazodone, mirtazapine, benzodiazepines, and Z-drugs) were detected in 61% (n = 25) of patients and were cross-tapered with daridorexant using the 25% x week rule, being totally dismissed within the first month. Other concomitant medications were not modified during the observation period.

Throughout treatment, we observed significant improvements in the ISI (F = 7.86; *p* = 0.002) and sTST (F = 6.22; *p* = 0.006) scores, without a significant interaction of any of the covariates with time (all *p* > 0.05) and with a mean difference (SE) in the ISI [−3.12 (0.164); −9.47 (0.38)] and in the sTST [0.34 (0.06); 1.05 (0.09)], respectively, after one and three months (*p* < 0.001 after Bonferroni correction of post hoc comparisons). Only between-subject differences regarding the overall clinical severity as measured by CGI-S scores had a significant effect on the sTST (F = 14.36; *p* = 0.002). All results from ANOVA for repeated measurements are summarized in Supplementary Table S1.

Quality of life and clinical global severity, anxiety and depression symptoms, and levels of craving improved at the endpoint. Mean changes from baseline in all secondary outcome measures were significant in the post hoc comparison after the Bonferroni correction, as reported in Table 2. Only baseline ISI scores displayed a significant interaction with changes over time in the HDRS (*p* = 0.004) and VASc scores (*p* = 0.048). Results with the effects of all the covariates on secondary outcome measures are summarized in Supplementary Table S2. All patients could be classified as at least “moderately ill” according to baseline CGI-S scores, and 51.2% of the initial sample was “markedly ill” (CGI-S scores = 5). At endpoint, only 14.6% of the sample was rated as “moderately ill” (CGI-S scores = 4), with the remaining patients being classified as either “mildly ill” (56.8%; CGI-S scores = 3) or “borderline ill” (28.6%; CGI-S scores = 2). Additionally, the patients had maintained abstinence by the end of treatment in 65.8% (n = 27) of cases.

At the endpoint, 4 patients (10.3%) were classified as “remitters”, 22 (53.8%) as “responders”, and 3 (7.7%) as “non-responders” as for ISI scores, while the dropout rate was 28.2% (n = 12). All dropouts occurred after the first month of treatment, and the main reasons for discontinuation were perceived suboptimal effects and other non-clinical reasons. A minority of patients (n = 8, 21.1%) reported mild side effects (i.e., drowsiness, sluggishness) that disappeared over time and did not lead to treatment discontinuation in any case.

Significant correlations were observed among the ISI, HARS, and HDRS scores as well as among the WHO-5, HARS, and HDRS scores at different time-points, while the ISI and VASc scores positively correlated at the endpoint. The main results from the correlations are shown in Table 3.

## 3. Discussion

In this report, we describe the effects of the new DORA daridorexant in patients with comorbid IDs and SUDs. We observed a significant improvement in insomnia and related symptoms after one month of treatment until the endpoint. Overall, more than half of the sample responded to treatment with daridorexant, with about 10% reaching complete remission of insomnia. SUD patients also displayed a reduction in craving throughout treatment and relapses into substance use were detected in less than half of the sample. Daridorexant was generally safe and well-tolerated; only a minority of patients reported mild side effects, which were transient and did not require treatment discontinuation. Accordingly, clinical studies indicate that daridorexant is generally well tolerated with an overall adverse event incidence comparable to placebo. Specifically, somnolence and headache have been reported as the most common adverse reactions, typically with mild-to-moderate intensity, and have not led to treatment discontinuation in long-term safety and tolerability trial reports [27,33,34].

Chronic insomnia is extremely frequent in SUDs with a prevalence up to three times higher than in patients with other psychiatric disorders [5]. Addressing sleep problems through substance use, especially of alcohol and cannabinoids, or the misuse of prescription anxiolytics/sedative–hypnotics, represents a common, dysfunctional self-medicating strategy [35,36]. According to a systematic review with meta-analysis, distinct patterns of association between specific sleep outcomes (e.g., impaired daytime functioning, evening chronotype) and substances can be identified, especially in young adults [35]. Although the sample size did not allow for a conclusive differentiation among SUD subtypes and their influence on treatment response, here, an exploratory analysis suggests the absence of significant associations between type of substances and treatment response (*p* = 0.712). Given the unbalanced distribution across substance categories, future studies with wider samples are needed to investigate predictors of responses to daridorexant and to determine whether specific SUDs might influence treatment outcomes. Nevertheless, this study is among the first to include patients with different diagnoses of SUDs, integrating previous findings primarily focused on opioid and anxiolytic/sedative–hypnotic use disorders [32,37].

It is noteworthy that the comorbidity between SUDs and other psychiatric diseases is generally burdened by a more severe course, with an overall high clinical severity and detrimental outcomes [38]. Further, sleep disruptions per se have been reported as significant predictors for the onset of depressive, anxiety, and psychotic symptoms [3] as well as, in SUDs, for the occurrence of craving, relapse risk, and additional psychopathology [7]. Here, almost half of the sample presented a psychiatric comorbidity, and relevant anxiety and depression symptoms were detected at baseline. Such symptomatology, although it did not exert a significant effect on the course of sleep measures, correlated with insomnia severity and patient-reported quality of life. Indeed, compared to the general population, the association with greater psychopathology and lower well-being has been described in subjects with mental illness and different sleep disturbances [39].

Consistent research findings have identified insomnia as a transdiagnostic process in psychiatric diseases, suggesting that treatments specifically targeting sleep alterations might exert beneficial effects on psychopathology [3,40]. Growing evidence describes the role of the orexin system in the pathophysiology of several psychiatric diseases [41]. Considering the overlapping neural circuits and the involvement in coordinating appropriate physiological/behavioral responses to stressful stimuli, alterations in orexin signaling have been hypothesized to play a role in disorders associated with reduced hedonic tone as well as with greater motivated arousal and anxiety, like depressive and anxiety disorders [42,43]. Accordingly, in this sample, psychopathological dimensions decreased at the endpoint within the improvement of overall clinical severity, with depression symptoms being significantly influenced by the initial severity of insomnia.

Interestingly, we observed reduced craving and a good abstinence rate after three months of treatment. Changes in craving significantly interacted with the baseline insomnia severity, and a positive correlation was also found between insomnia severity and craving at the endpoint, while a trend could be detected at baseline (*p* = 0.06). These observations are in line with previous reports showing a reciprocal influence between sleep disturbances and levels of craving [44,45]. Thus, it could be hypothesized that improvements in insomnia might contribute to abstinence maintenance, as well as to better clinical outcomes.

Orexins can enhance feelings of pleasure and reward from different stimuli, which results in the improvement of hedonic behaviors, and dynamic shifts in orexin production can contribute to adaptive motivational regulation [19]. This mechanism can be dysfunctional in psychiatric disorders characterized by motivational impairments, such as SUDs, where aberrant, persistently high expression in orexin levels may lead to focused hyper-motivation for substances [20]. In SUDs, the overactivation of this system has been hypothesized to contribute to several addiction-related behaviors, like drug-seeking and withdrawal [20]. In turn, substance use seems to induce maladaptive changes in the orexin pathway, sustaining drug intake and promoting relapses [46].

The orexin system has been proposed as a target for novel pharmacological interventions in psychiatric disorders, and its involvement in mechanisms regulating motivation makes it an interesting target also for the addiction field [46,47,48]. Preliminary evidence has been collected about the positive effects of orexin receptor antagonism on stress-induced relapses and drug-seeking behavior in different kinds of SUDs [37,49,50,51,52,53]. Among these antagonists, daridorexant stands out for its dual action on orexin receptors type 1 and 2, which not only promotes sleep but may also attenuate the hyperactivation of reward pathways associated with substance use [21,22]. Specifically, preclinical research has found increased cocaine self-administration following orexin peptide assumption, in contrast with reduced motivation for substance and cocaine-associated impulsiveness induced by orexin antagonists [52]. As well as this, the helpfulness of orexin neurotransmission-modulating compounds has been suggested in managing craving and withdrawal symptoms in opioid-use disorder [37] and nicotine dependence [50]. In line with this evidence, daridorexant’s dual orexin receptor antagonism may contribute to the reduction in craving observed in our study, further supporting its potential role in the management of addiction-related behaviors.

Aside from the therapeutic effect of orexin modulators on clinical manifestations of psychiatric diseases, the overall benefit of improving sleep disturbances in these patients remains indisputable, and insomnia should be treated as a problem of its own in mental health services [3]. Specific non-pharmacological interventions, such as cognitive behavioral therapy for insomnia (CBT-I), have shown efficacy in improving sleep measures as well as substance-related problems in patients seeking treatment for SUDs [36,54]. However, in patients with comorbid SUDs, CBT-I techniques are often delayed until abstinence is established, and feasibility issues are frequently detected [54,55].

Despite the high co-occurrence rates of IDs and SUDs, effective therapeutic options for these patients are limited, and the remission of insomnia often remains an unmet need, with negative implications for daytime functioning and full functional recovery [4]. In our study, baseline assessments indicated a substantial clinical burden, with most patients classified as at least moderately ill and over a half rated as markedly ill. By the end of treatment, both clinical severity and self-reported quality of life showed meaningful improvements, albeit without significant associations between these dimensions. This may suggest that reductions in clinical severity do not necessarily correspond to proportional gains in subjective well-being or that these dimensions follow distinct trajectories over time. However, it is noteworthy that the limited sample size may have prevented us from detecting a significant relationship, highlighting the need for further research with larger cohorts and a more comprehensive assessment of real-life functioning. Nevertheless, our findings provide further evidence supporting the role of DORAs as promising therapeutic agents in this clinical population, particularly daridorexant, which is currently the only compound within this pharmacological class to have received approval from both the European and the Italian regulatory authorities [27,56].

The main limitations of this study include its retrospective design and lack of objective sleep measurements (e.g., actigraphy, polysomnography) assessing duration, latency, quality, and efficacy, as well as daytime sleepiness. The small sample size and absence of a control group (e.g., placebo, alternative intervention, or ID patients without SUDs) limited both the ability to isolate daridorexant’s specific effects from possible confounders (e.g., concurrent pharmacological treatments) and to evaluate potential response predictors, including substance use type, SUD severity, or other psychopathological factors. Additionally, generalizability to broader settings remains uncertain, as data were derived from help-seeking outpatients at a single center, with potential selection bias due to the exclusion of a substantial number of screened participants. Future studies with larger, more diverse, and multi-center samples are needed to confirm the applicability of these findings.

## 4. Materials and Methods

### 4.1. Participants

Data were extracted from medical charts of outpatients consecutively referred for substance-related problems to the “Centro Psichiatrico Integrato per la ricerca, la prevenzione e la cura delle Dipendenze” (CePID), Department of Psychiatry, of Fondazione Policlinico Universitario Agostino Gemelli IRCCS, in Rome, between December 2022 and January 2024. The inclusion criteria were as follows: an age ≥ 18 years; fluency in spoken and written Italian; a diagnosis of an ID according to the DSM-5-TR criteria (APA, 2022), rated as moderate-to-severe with an Insomnia Severity Index (ISI) score ≥ 15 [57]; abstinence from alcohol and other substances at the time of assessment and for at least the previous 3 months, based on the TimeLine Follow Back calendar, blood alcohol content levels, and urinary toxicological tests; and the prescription of 50 mg/day of daridorexant, introduced either directly or after a cross-taper with other pharmacological treatments for insomnia ongoing at baseline, using the 25% x week rule. The presence of other concomitant psychopharmacological medications (i.e., anticonvulsants/mood stabilizers, antidepressants, antipsychotics, anti-craving drugs, and anxiolytics) was not a criterion for exclusion, provided that these were not modified throughout the observation period. The exclusion criteria were as follows: psychotic features; organic brain syndromes; and neurocognitive disorders or cognitive impairment based on a Mini-Mental State Examination score < 26 [58]. Anonymity was guaranteed to all patients, who gave written informed consent for enrollment in this study.

### 4.2. Data Collection

Sociodemographic (age, gender, educational level, occupation, marital status) and clinical data (Body Mass Index, family history of mental illnesses, medical and psychiatric comorbidities, smoking habits) were collected. Trained clinicians evaluated the presence of SUDs and other psychiatric disorders through the Structured Clinical Interview for DSM-5 Disorders—Clinician Version (SCID-5-CV) and for Personality Disorders (SCID-5-PD), respectively.

Insomnia was assessed by its duration (months), typology (initial, middle, late, mixed), and severity through the ISI score (≥15 and <22, moderate; ≥22, severe) at baseline and after one and three months of treatment. At the endpoint, patients were classified as “remitters” if the ISI score was <8, whereas reductions in the baseline score of ≥8 and <8 points were used to rate participants as, respectively, “responders” and “non-responders”. At the same time-points, the average subjective total sleep time (sTST) was derived from a sleep diary that patients completed daily over the previous two weeks. Bedtime, wake-up time, sleep onset latency, and nighttime awakenings were recorded and incorporated into a specific formula to calculate the sTST [59]. To ensure data reliability, participants received both written and verbal instructions, including practical examples, and were encouraged to complete the diary each morning to minimize recall bias [60].

Clinicians administered the Hamilton Anxiety Rating Scale (HARS), the Hamilton Depression Rating Scale (HDRS) [61,62], and the Clinical Global Impression Severity Scale (CGI-S) [63] at baseline and at the endpoint to investigate the presence and the intensity of anxiety and depressive symptomatology and to rate the overall severity of psychiatric symptoms. At the same time-points, self-perceived changes in overall quality of life were evaluated through the 5-item World Health Organization Well-Being Index (WHO-5) [64], while craving for substances was investigated with the Visual Analog Scale for Craving (VASc) [65] and abstinence was concurrently monitored. Patients were considered abstinent if they maintained sobriety and did not consume any substances—regardless of the amount—throughout the observation period; otherwise, a relapse was recorded [66,67].

### 4.3. Statistical Analysis

Descriptive data were summarized as the number of patients and percentage (%) or mean ± standard deviation (M ± SD) for categorical and continuous variables, respectively. The outcome measures—mean changes from baseline to different time-points in each variable—were analyzed using ANOVA for repeated measurements, with specific covariates for the primary (i.e., age, gender, psychiatric comorbidities, anxiety and depression symptoms, overall clinical severity) and the secondary outcomes (i.e., age, gender, baseline insomnia severity). The Bonferroni procedure was applied as a post hoc test following the analyses of variance. The relationship between sleep measures, anxiety and depression symptoms, craving, and quality of life was investigated through the Pearson correlation, after controlling for the parametric/non-parametric distribution of variables. A significance level of 0.05 was used for each test. All analyses were conducted using IBM SPSS Statistics for Windows, v. 28.0 (IBM Co., Armonk, New York, NY, USA).

The sample size was determined based on a statistical power analysis, a review of the literature, and the recruitment capacity of the center. A sensitivity analysis using G*Power (a flexible statistical power analysis program for biomedical sciences, as described by Faul et al.) [68] indicated that with n = 34, there would be an 80% power (1 − β = 0.80) to detect a minimally relevant effect size of δ ≥ 0.5, assuming a two-sided test with a Type I error rate of α = 0.05. Considering an expected 15% dropout rate, a final sample size of at least 39 participants was estimated.

## 5. Conclusions

Given the worldwide spread of insomnia, investigating all risk factors and recognizing the specificity of different clinical samples is necessary to identify and deliver personalized treatments. Careful assessments and combined interventions aimed at managing both IDs and SUDs may be a valuable therapeutic strategy in comorbid samples. In this study, we observed a significant improvement in insomnia following a 3-month treatment with daridorexant in SUD patients. The benefits of using orexin receptor antagonists in SUDs might derive from a combined effect on orexin regulation of both top–down (i.e., sleep) and bottom–up (i.e., motivation) pathways [69]. Given the peculiar and innovative mechanism of action, the good tolerability and low abuse liability, and its potential role in addressing symptoms such as depression, anxiety, and stress-related manifestations, as well as craving, daridorexant might represent a promising therapeutic approach in the field of addiction. Future studies with larger and more balanced samples, as well as controlled designs, are needed to better investigate predictors of response to daridorexant and to determine whether specific patient characteristics might influence treatment outcomes.

## Figures and Tables

**Table 1:** Sociodemographic and clinical characteristics at baseline.

*Characteristics* (*n*, %; *M* ± *SD*)	
**Overall**	41
*Sociodemographic features*	
**Age** (years)	50.4 ± 13.7
**Gender**	
Male	26 (63.4)
Female	15 (36.6)
**Educational level** (years)	13.8 ± 2.8
High school	16 (39)
**Occupation**	
Employed	30 (73.2)
Unemployed	11 (26.8)
**Marital status**	
Married	13 (31.7)
Unmarried	20 (48.8)
Divorced	8 (19.5)
*Clinical data*	
**BMI**	24.4 ± 5.2
**Smoking habits** (yes)	23 (56.1)
**Medical comorbidities** (yes)	29 (70)
**Psychiatric comorbidities** (yes)	20 (48.8)
**Family psychiatric history** (yes)	19 (47.5)
**Insomnia duration** (months)	17.4 ± 8.7
**Insomnia severity #**	
Moderate	25 (61)
Severe	16 (39)
**Insomnia typology**	
Initial	13 (31.7)
Middle	4 (9.8)
Late	1 (2.4)
Mixed	23 (56.1)
*Psychometric assessment*	
**CGI-S**	4.5 ± 0.5
**HARS**	17.7 ± 2.7
**HDRS**	15.5 ± 5.1
**ISI**	19.9 ± 3.2
**sTST**	5.08 ± 0.6
**VASc**	4.42 ± 1.8
**WHO-5**	10.5 ± 1.6

Abbreviations: #, according to ISI cut-off scores; BMI, Body Mass Index; CGI-S, Clinical Global Impression Severity Scale; HARS, Hamilton Anxiety Rating Scale; HDRS, Hamilton Depression Rating Scale; ISI, Insomnia Severity Index; *M*, mean; SD, standard deviation; sTST, subjective total sleep time; VASc, Visual Analog Scale for Craving; WHO-5, 5-item World Health Organization Well-Being Index.

**Table 2:** Changes from baseline to endpoint in clinical global severity, anxiety and depression symptoms, craving, and quality of life.

*Mean Change from Baseline (SE)*		*p*
**CGI-S**	−1.6 (0.30)	0.001
**HARS**	−1.8 (0.26)	<0.001
**HDRS**	−1.7 (0.38)	<0.001
**VASc**	−1.3 (0.15)	<0.001
**WHO-5**	3.4 (0.16)	<0.001

Abbreviations: CGI-S, Clinical Global Impression Severity Index; HARS, Hamilton Anxiety Rating Scale; HDRS, Hamilton Depression Rating Scale; *p*, statistical significance; SE, standard error; VASc, Visual Analog Scale for Craving; WHO-5, 5-item World Health Organization Well-Being Index.

**Table 3:** Correlations between sleep measures, anxiety and depression symptoms, craving, and quality of life at different time-points.

	*ISI*		*HARS*		*HDRS*	
**Pearson’s r (*p* Value)**	*Baseline*	*3 Months*	*Baseline*	*3 Months*	*Baseline*	*3 Months*
**ISI**						
*Baseline*	-	-	0.32 (0.07)	0.41 (0.04) *	0.53 (0.002) *	0.64 (<0.001) *
*3 months*	0.81 (<0.001) *	-	0.31 (0.15)	0.41 (0.05) *	0.42 (0.04) *	0.51 (0.013) *
**VASc**						
*Baseline*	0.28 (0.12)	0.29 (0.15)	−0.10 (0.63)	0.01 (1)	0.05 (0.83)	0.12 (0.59)
*3 months*	0.36 (0.06)	0.41 (0.039) *	−0.03 (0.89)	0.08 (0.71)	0.09 (0.66)	0.09 (0.65)
**WHO-5**						
*Baseline*	−0.14 (0.43)	−0.06 (0.77)	−0.29 (0.11)	−0.52 (0.008) *	−0.42 (0.02)*	−0.55 (0.006) *
*3 months*	−0.13 (0.53)	−0.02 (0.93)	−0.31 (0.14)	−0.36 (0.09)	−0.36 (0.09)	−0.39 (0.06)

* Significant results. Abbreviations: HARS, Hamilton Anxiety Rating Scale; HDRS, Hamilton Depression Rating Scale; ISI, Insomnia Severity Index; *p*, statistical significance; r, coefficient; VASc, Visual Analog Scale for Craving; WHO-5, 5-item World Health Organization Well-Being Index.

## Data Availability

The authors do not have permission to share data.

## Supplementary Materials

The following supporting information can be downloaded at: https://www.mdpi.com/article/10.3390/ph18030378/s1, Table S1. Changes in sleep measures at different time-points by age, gender, psychiatric comorbidity, anxiety and depression symptoms and overall clinical severity as covariates. Table S2. Changes from baseline in clinical global severity, anxiety and depression symptoms, craving and quality of life by age, gender, and baseline insomnia severity as covariates.
