# Supplementary material for: A Preliminary Report on the Effects of Daridorexant in Patients with Comorbid Insomnia and Substance Use Disorders
Source: Pharmaceuticals (Basel). 2025 Mar 6;18(3):378. doi: 10.3390/ph18030378 (PMC11946062; doi:10.3390/ph18030378)
Supplement: Supplementary file 1 [file pharmaceuticals-18-00378-s001.zip › Table S1.pdf]

Supplementary Table S1. Changes in sleep measures at different time-points by age, gender, psychiatric comorbidity, anxiety and depression symptoms and overall clinical severity as covariates.

**ISI**	F	*p*
Within Subjects Effects		
**Time**	7.86	**.002**
**Time * Age**	0.17	.841
**Time * Gender**	0.27	.767
**Time * Group[1]**	1.04	.365
**Time * Group[2]**	0.26	.772
**Time * Group[3]**	0.91	.416
**Time * Group[4]**	2.68	.085
Between Subjects Effects		
**Age**	0.05	.834
**Gender**	3.31	.089
**Group[1]**	2.52	.133
**Group[2]**	0.89	.360
**Group[3]**	1.01	.333
**Group[4]**	3.83	.069

**sTST**		
Within Subjects Effects		
**Time**	6.22	**.006**
**Time * Age**	0.47	.631
**Time * Gender**	3.15	.058
**Time * Group[1]**	1.47	.247
**Time * Group[2]**	0.46	.634
**Time * Group[3]**	1.63	.213
**Time * Group[4]**	3.03	.064
Between Subjects Effects		
**Age**	0.72	.413
**Gender**	0.04	.840
**Group[1]**	0.17	.689
**Group[2]**	1.67	.218
**Group[3]**	0.01	.960
**Group[4]**	14.3	**.002**

Significant results in **bold**. Abbreviations: F, between- and within-group ratio; Group[1], Psychiatry comorbidity; Group[2], Anxiety symptoms (HARS scores); Group[3], Depression symptoms (HDRS scores); Group[4], Global clinical severity (CGI-S scores); ISI, Insomnia Severity Index; sTST, subjective Total Sleep Time; *p*, statistical significance.
