# Supplementary material for: A Preliminary Report on the Effects of Daridorexant in Patients with Comorbid Insomnia and Substance Use Disorders
Source: Pharmaceuticals (Basel). 2025 Mar 6;18(3):378. doi: 10.3390/ph18030378 (PMC11946062; doi:10.3390/ph18030378)
Supplement: Supplementary file 1 [file pharmaceuticals-18-00378-s001.zip › Table S2.pdf]

Supplementary Table S2. Changes from baseline in clinical global severity, anxiety and depression symptoms, craving and quality of life by age, gender, and baseline insomnia severity as covariates.

**CGI-S**	F	*p*
Within Subjects Effects		
**Time * Age**	1.27	.302
**Time * Gender**	0.01	.911
**Time * Group[1]**	3.47	.112
Between Subjects Effects		
**Time * Age**	1.15	.325
**Time * Gender**	5.74	.054
**Time * Group[1]**	1.62	.250

**HARS**		
Within Subjects Effects		
**Time * Age**	0.31	.581
**Time * Gender**	0.08	.781
**Time * Group[1]**	0.15	.703
Between Subjects Effects		
**Time * Age**	0.42	.524
**Time * Gender**	0.22	.644
**Time * Group[1]**	3.23	.087

**HDRS**	F	*p*
Within Subjects Effects		
**Time * Age**	0.17	.841
**Time * Gender**	0.27	.767
**Time * Group[1]**	1.04	.365
Between Subjects Effects		
**Time * Age**	0.35	.561
**Time * Gender**	0.06	.802
**Time * Group[1]**	10.4	**.004**

**VASc**		
Within Subjects Effects		
**Time * Age**	1.74	.199
**Time * Gender**	0.85	.364
**Time * Group[1]**	0.74	.397
Between Subjects Effects		
**Age**	0.34	.566
**Gender**	0.03	.864
**Group[1]**	4.34	**.048**

**WHO-5**		
Within Subjects Effects		
**Time * Age**	0.02	.879
**Time * Gender**	0.02	.881
**Time * Group[1]**	3.13	.091
Between Subjects Effects		
**Age**	0.05	.816
**Gender**	0.01	.882
**Group[1]**	0.99	.330

Significant results in **bold**. Abbreviations: CGI-S, Clinical Global Impression-Severity Index; F, between- and within-group ratio; Group[1], Insomnia Severity at baseline (ISI scores); HARS, Hamilton Anxiety Rating Scale; HDRS, Hamilton Depression Rating Scale; *p*, statistical significance; VASc, Visual Analogue Scale for craving; WHO-5, 5-item World Health Organization Well-Being Index.
